# Household non-thermal processing for the dissolution of sea buckthorn flavonoids and the effects on its bioactive characteristics

**DOI:** 10.1016/j.fochx.2025.102941

**Published:** 2025-08-21

**Authors:** Jie Sheng, Lanlan Yao, Qingying Dong, Bin Zhou, Bin Li, Guoqiang Zhang, Hongshan Liang

**Affiliations:** a College of Food Science and Technology, Huazhong Agricultural University, Wuhan, Hubei 430070, PR China; b Wuhu Green Food Industry Research Institute Co., Ltd., Wuhu 241000, China; c Key Laboratory of Fermentation Engineering, Ministry of Education; National “111” Center for Cellular Regulation and Molecular Pharmaceutics; Hubei Key Laboratory of Industrial Microbiology; School of Biological Engineering and Food, Hubei University of Technology, Wuhan 430068, China; d Key Laboratory of Environment Correlative Dietology, (Huazhong Agricultural University), Ministry of Education, Wuhan, Hubei 430070, PR China

**Keywords:** Non-thermal food processing, Sea buckthorn flavonoids, Hydrophobic flavonols, Dissolution kinetics, Bioactivity

## Abstract

Sea buckthorn flavonoids exhibit high utilization value in the processing of functional foods and non-thermal processing (NTP) has emerged as a sustainable alternative for flavonoid extraction. Among total flavonoid content evaluation by juicing, shearing, ultrasound, colloid mill, and high-pressure homogenization, juicing demonstrated comparable efficiency. Systematic characterization of sea buckthorn flavonoids/flavonols dissolution (SBF) revealed that its color, turbidity, and particle size progressively changed during processing, flavonol releasement exhibited time-dependent patterns, with cumulative dissolution rates reaching 14.15 % (quercetin), 12.79 % (kaempferol), 6.75 % (isorhamnetin) within 180 min. Fitting with first-order kinetics and Weibull model suggested that the dissolution mechanisms involved both diffusion-controlled and erosion-mediated. Besides, sea buckthorn dissolution products (SBD) showed strong α-glucosidase inhibition (IC_50_: 5.18 mg/mL) and oxygen radical absorbance capacity (ORAC: 428.76 μmol TE/mL). This study elucidated the dissolution behavior of SBF under NTP conditions, with particular focus on the kinetics of flavonol release, providing guidance for future value-maximizing utilization of SBF.

## Highlights

• No significant difference in total SBF dissolution between household and industrial juicing.
• Flavonols released rapidly within 20 min, achieving 6.75–14.15 % cumulative dissolution in 180 min.
• Three flavonols followed the diffusion-erosion composite mechanism.
• Flavonoids released from juicing exhibited potent α-glucosidase inhibition and high ORAC.

## Graphical abstract

**Figure:**
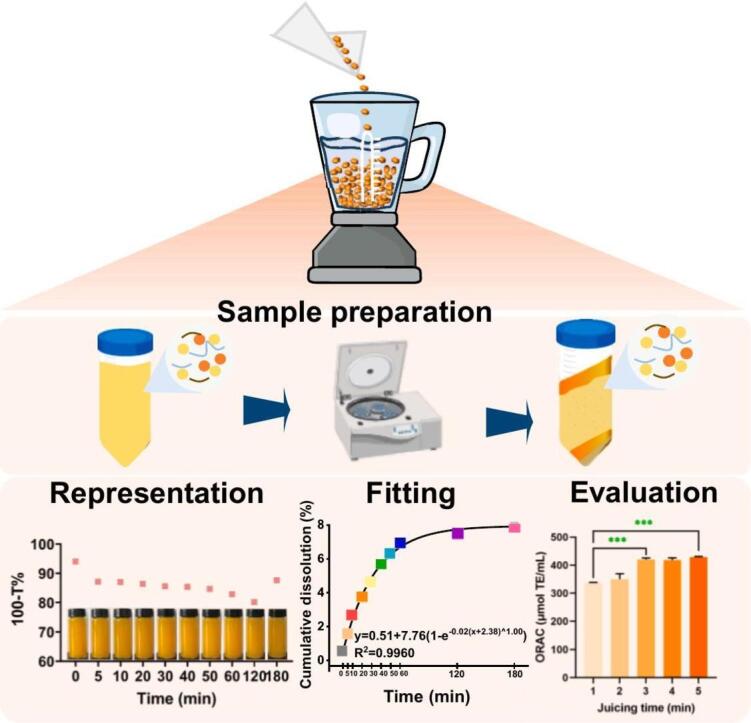

## 1 Introduction

Sea buckthorn (*Hippophae rhamnoides* L.) (SB), a dual-purpose medicinal-food plant, has garnered significant research interest due to its unique phytochemical profile containing flavonoids, vitamins and polyunsaturated fatty acids (Ciesarova et al., 2020; Ma et al., 2022). Notably, SB flavonoids (SBF) exhibit multifaceted pharmacological activities including antioxidant, anti-inflammatory and anti-tumor effects (Xu et al., 2024). However, their dissolution efficiency is constrained by two critical factors: glycosidic conjugation within cell wall matrices requiring structural disruption for liberation (Shahidi & Yeo, 2016); inherent hydrophobicity of some flavonoids leading to aqueous phase insolubility and recrystallization tendencies (Premathilaka et al., 2022). Therefore, the bioavailability limitation of these compounds restricts their development and application in the food and pharmaceutical fields. While conventional solvent extraction improves yields, residual solvent risks necessitate safer alternatives.

The liberation of bioactive compounds from plant tissues requires mechanical intervention to compromise structural integrity. Modern fruit and vegetable processing operations predominantly employ either thermal or non-thermal processing (NTP) technologies. While thermal processing (e.g., cooking, spray drying) extends shelf life by microbial inactivation, it compromises bioactive stability and sensory quality (Ursache et al., 2017). NTP, in contrast, achieve microbial safety while better preserving functional and sensory properties, enhancing bioactive release via cel wall modulation (Kumar et al., 2021; Liu et al., 2024). For instance, high-pressure processing (HPP) uses a hydrostatic pressure of 100 to 1000 MPa to disrupt the membrane bilayers and induce an increase in cell permeability. Recent studies have shown that 500 MPa/5 min treatment of SB juice elevated phenolic content to 374.48 mg GAE/100 mL (Xia et al., 2023). Ultrasound-assisted extraction (UAE) employs cavitational shear to fracture cellulose networks, optimizing polyphenolic compounds yields within 30 min pulsed operation (Pap et al., 2024). Besides, pulsed electric fields (PEF) (1–10 kV/cm) induce electroporation, synergistically enhancing juice yield by 11.37 % when combined with HPP (Zhang et al., 2024). Despite technological advances, industrial scale NTP faces economic viability challenges due to high capital costs and batch limitations (Picart-Palmade et al., 2019). While, on the consumer perspective, active compounds study of household scale NTP remains notably underdeveloped and still needs more attention.

The global juicer market size was estimated at USD 2.65 billion in 2023 and is expected to grow at a CAGR of 7.4 % from 2024 to 2030 (https://www.grandviewresearch.com/industry-analysis/juicer-market-report), reflecting sustained consumer demand for household juicing tools and underscoring their critical role in nutrient acquisition. Despite this growing interest in functional food preparation methods, existing studies on non-thermal processing of sea buckthorn have primarily focused on industrial-scale technologies (e.g., high-pressure homogenization, ultrasonic extraction) to optimize bioactive retention, with limited attention to domestic non-thermal methods (e.g., household juicing). While household processing is widely adopted by consumers, the mechanisms by which simple mechanical disruption (e.g., juicing time) affects the release kinetics of hydrophobic flavonoid aglycones and their subsequent bioactivity remain underexplored. Specifically, there is a lack of data linking physical changes in fruit matrices (e.g., particle size, microstructure) induced by domestic processing to the dissolution behavior of these flavonoids and their functional properties. This gap hinders our understanding of how household processing can be optimized to maximize the nutritional value of sea buckthorn for daily consumption. Besides, mathematical modeling has emerged as an indispensable approach for deciphering complex phytochemical release mechanisms in food processing. Established models including first-order kinetics, Weibull, Korsmeyer-Peppas, and Higuchi, enable mechanistic analysis through parameter optimization against dissolution profiles. For instance, Benítez-Correa et al. identified optimal models for flavonoid release from cocoa husks by comparing first-order kinetics with biphasic diffusion models (Benítez-Correa et al., 2025). Similarly, the deglycosylation kinetics of genistein were shown to obey first-order principles (Chen et al., 2023), while Zhou et al. elucidated mass transfer mechanisms in hesperidin extraction through kinetic modeling (Zhou et al., 2023). These advancements highlight the transformative potential of mathematical frameworks in bridging empirical observations with mechanistic insights.

This study evaluated five mechanical disruption methods (household juicing, shear processing, ultrasonication, colloid milling, high-pressure homogenization) for SB total flavonoid content (TFC). Subsequent juicing process-based investigations characterized the physical characteristics (macroscope feature, turbidity, UV–Vis, pomace particle size distribution, and microstructure) and electronic sensory characteristic, analyzed time-dependent dissolution kinetics of target flavonols (quercetin, kaempferol and isorhamnetin) through first-order and Weibull modeling. The correlation between SB juice biological efficacy (α-glucosidase inhibition ability and oxygen free radical absorption capacity (ORAC)) and released flavonols concentration was assessed via the principal component analysis (PCA). Finally, the stability of SBF was evaluated through storage experiment. We hypothesize that varying the duration of household juicing (a representative domestic non-thermal mechanical process) will modulate the physical disruption of SB matrices, thereby affecting the release efficiency of hydrophobic flavonoid aglycones. Specifically, prolonged juicing time will enhance matrix breakdown (smaller particle size, disrupted microstructure), leading to increased cumulative release of these aglycones, which in turn will correlate with improved in vitro bioactivity (higher antioxidant capacity and α-glucosidase inhibitory activity). This work bridged critical knowledge gaps in household NTP applications and provided a theoretical basis for the intake of nutritional factors in the daily dietary guidelines of the family.

## 2 Material and method

### 2.1 Materials and reagents

SB fruit was purchased from Chifeng Inner Mongolia.

Sea buckthorn (*Hippophae rhamnoides L.*) originates from Chifeng, Inner Mongolia, China. It is a wild primitive variety named “Xiaoguo Shaji” cultivated in the Hunshandake Sandy Land. The fruits are round, mainly in colors of yellow, orange-yellow, and light red, and are commonly used in functional food research. Quercetin, kaempferol, isorhamnetin, acetonitrile, methanol, and ethyl acetate were all HPLC grade (≥98 %) and obtained from Thermo Fisher Scientific (Waltham, MA, USA). Distilled water and ultra-pure water were used in the experiment. Other regents were purchased from Aladdin Biochemical Technology Co., LTD (Shanghai, China).

### 2.2 Determination of physical and chemical properties

The soluble solid content (SSC) of SB was determined by hand refractometer. Briefly, the hand refractometer was calibrated to zero with distilled water. Then the puree supernatant was dropped onto the hand refractometer and the results were expressed in °Brix. The titrable acidity (TA) was measured by acid-base titration (Rezvani & Goli, 2024). The volume of NaOH consumed was recorded immediately when the indicator liquid changed from transparent to red, and the TA of SB pulp was calculated according to the consumed NaOH. Moisture content (MC) was determined by water activity meter. Total sugar content (TSC) was calculated by anthrone sulfuric acid process (Wang et al., 2020) and pH was measured using pH meter. The experiment was repeated three times.

### 2.3 Preparation of sea buckthorn dissolution products (SBD)

#### 2.3.1 Juicing

Appropriate amount of undamaged SB fruits was surface-sanitized and dehydrated prior to homogenization with distilled water. Mechanical processing was conducted using a laboratory-scale juicer (L6-L621, Joyoung, China) (1000 rpm) with variable durations (1–5 min). The resultant slurry underwent immediate centrifuged at 8000 rpm for 10 min (H1850R, cence, China) to separate solid phase and liquid phase. Parenchyma tissue retention was used to physical characterization and liquid phases were used to flavonoid concentration determination. The experiment was repeated three times.

#### 2.3.2 Shearing

Freshly harvested SB fruits underwent primary mechanical disruption using a juicer. Precisely aliquoted samples were subsequently subjected to high shear processing (T25 Digital, IKA, German) (12,000 rpm) with temporal gradients (1–5 min) in an emulsion cup. Post-treatment fractionation involved dual pathways: immediate parenchymal tissue preservation for microstructural analysis and centrifugal phased separation (8000 rpm, 10 min) for supernatant acquisition. The experiment was repeated three times.

#### 2.3.3 Ultrasound

Following initial mechanical disruption, SB fruits were subjected to ultrasonic treatment which was carried out using an ultrasonic machine (SCIENTZ-IID, SCIENTZ, China) operating at 1000 W, 25 °C for 10 min, 20 min, 40 min, 60 min, and 120 min, respectively. Post-ultrasonic treatment, partially SB pulp was preserved for following analysis and others were centrifuged and collected supernatant at 8000 rpm for 10 min. The experiment was repeated three times.

#### 2.3.4 Colloid mill

Initially crushed fresh fruits were filtered through a 100-mesh screen to remove seeds and large particles of pulp, and then grind by colloid mill (JML-50, KELAO ENTERPRISE, China) for 10, 20, 30, 40 and 50 min, respectively. The obtained pulp was centrifuged (8000 rpm, 10 min) and the supernatant was collected for further investigation. The experiment was repeated three times.

#### 2.3.5 High pressure cell homogenizer

The pulp obtained by 100-mesh sieve was homogenized and crushed by a high pressure cell homogenizer (AH-2010, ATS, Canada), the pressure was set at 30 MPa, 50 MPa, 70 MPa, 90 MPa and 110 MPa. The pulp was treated for 10 min at each pressure. The result slurry of high-pressure homogenization was centrifuged (8000 rpm, 10 min) and collected supernatant and sediment respectively for subsequent experiments. The experiment was repeated three times.

### 2.4 Chemical extraction of flavonols

#### 2.4.1 Flavonol extraction from pulp supernatant

The supernatant obtained after removing the pulp from the mixture was designated as fruit juice, which consisted of a juice layer and an oil phase. An equal amount of ethyl acetate was added to the juice. The mixture was vortexed at 300 rpm for 5 min to fully extract compounds. After extraction, the organic phase was separated by centrifugation at 8000 rpm for 10 min. The ethyl acetate was dried by pressure blowing concentrator (MTN-2800D, AUTO SCIENCE, Canada), and then the equal amount of methanol was added to redissolve, the obtained extraction was detected by high-performance liquid chromatography diode-array detector (HPLC-DAD) after passing through a 0.22 μm filter.

#### 2.4.2 Flavonol extraction from fruit pomace

The solid fraction of pulp was collected by centrifugation. The extraction method of flavonols was according to the China national standard NY/T 3950–2021. In short, 5 g (exact value 0.001 g) of SB pomace was weighed into a 100 mL round-bottom flask, and 80 mL ethanol-hydrochloric acid (7.5:1, *v*/v) was added, then extracted at 90 °C by condensing reflux device for 90 min. After cooling, the mixture was transferred to a 100 mL volumetric flask, titrated to 100 mL by ethanol-hydrochloric acid, and centrifuged at 4000 rpm for 10 min. The resulting supernatant was analyzed by HPLC after passing through a 0.22 μm filter. Flavonol content was calculated by the Eq. 1:

(1) $$W=C^{*}V/m^{*}F$$

where, W represents the sample content (μg/g); C represents the three flavonols (quercetin, kaempferol, isorhamnetin) concentration of sample (μg/mL) obtained by three standard curves (Table S1); V represents the final volume of extraction solution (mL); m represents sample weight (g); F represents the dilution factor of sample extract.

### 2.5 Determination of TFC

TFC of samples was determined according to the previous study with some modification (Ge et al., 2022). Briefly, 20 μL redissolved extract solution was diluted by 100 μL Milli-Q water and mixed with 6 μL 5 % NaNO_2_ for 6 min, then 6 μL 10 % AlCl_3_•6H_2_O was mixed for 5 min, and finally 40 μL NaOH was added and mixed. The absorbance of the sample was measured at 510 nm. TFC were expressed as milligrams of catechin equivalent (CE) mL of liquid extracts.

### 2.6 Determination of flavonols content

Flavonols were detected using HPLC (e2965, Waters Corporation, USA) according to previous report with slight modifications (Mikulic-Petkovsek et al., 2012). The separation of three flavonols was performed on an Innoval ODS-2 C18 column (4.6✕250 mm, 5 μm). Mobile phase composition (solvent A: 0.6 % phosphoric acid in water; solvent B: acetonitrile) and gradient elution program: 0–10 min, 67–68 % A; 10–35 min, 68–69 % A; flow rate: 1 mL/min; injection volume: 10 μL; detection wavelength: 360 nm for flavonols; column temperature: 30 °C. The extracted sample solutions were filtered through a 0.22 μm polyethersulfone filter. SBF (quercetin, kaempferol, isorhamnetin) were identified by comparison of retention times with authentic standards under identical chromatographic conditions, quantified based on the external standard curves of quercetin (R^2^ = 0.9998), kaempferol (R^2^ = 0.9993), and isorhamnetin (R^2^ = 1) (Table S1). The LOD value of quercetin, kaempferol, and isorhamnetin was 0.015 μg/mL, 0.003 μg/mL, and 0.03 μg/mL, respectively. The LOQ value of quercetin, kaempferol, and isorhamnetin was 0.05 μg/mL, 0.01 μg/mL, and 0.1 μg/mL, respectively.

### 2.7 Parameter of puree

#### 2.7.1 Morphology

Morphology of samples were regularly recorded using a digital camera. The microstructure of SB pomace was characterized by scanning electron microscopy (SEM) (SU3800, Hitachi, Japan). Briefly, the pomace was dried and pretreated through a 100-mesh sieve. Subsequently, the resulting powder was evenly spread on double-sided adhesive tape using a toothpick. The pomace morphology was observed at magnifications of 150× and 1000 × .

#### 2.7.2 Turbidity

Sample turbidity was determined by turbidimetry using a microplate reader. In brief, the supernatant of samples was diluted in a certain proportion and measured absorbance value at 600 nm using a microplate, and the turbidity of the sample was calculated by the following formulas (Eq. 2 and Eq. 3):

(2) $$\text{Transmission rate}\;\left(T\%\right)={10^{-A}}^{*}100$$

(3) $$\mathrm{Turbidity}\;\left(\%\right)=100-T\%$$

where, A is the absorbance value of samples.

#### 2.7.3 Colorimetric analysis

The chromatic properties of samples were quantitatively evaluated using a colorimeter (Ultroscan VIS, HunterLab, USA), where L*, a*, and b* values represent lightness, red-green tone, and blue-yellow tone, respectively. After calibration, samples were poured into quartz cuvettes; chroma differences were calculated via formula and expressed as total color change (ΔE*) following Rezvani and Goli (2024). Additionally, samples in quartz cuvettes were tested for transmittance at 600 nm using a UV–Vis spectrophotometer.

(4) $${\mathrm{\Delta E}}^{*}=\sqrt{{\left(L-L_{0}\right)}^{2}}+\sqrt{{\left(a-a_{0}\right)}^{2}}+\sqrt{{\left(b-b_{0}\right)}^{2}}$$

where the “0” represents the color value of the control group.

#### 2.7.4 UV–Vis

The UV full wavelength scanning of SBD was determined using a microplate reader. SBD extracted solution was added to the microplate and absorption spectra were recorded in the range 200–500 nm. The intensity and distribution of ultraviolent absorption peaks of flavonols were analyzed through UV–Vis spectrum diagram.

#### 2.7.5 Particle size of SB pomace

The particle size of pomace was determined using the laser particle size distribution instrument (APA2000, MALVERN, UK). The pomace was diluted with Mili-Q water to a laser intensity of 5–10 % and then drew the particle size distribution curve. Samples were determined for three times.

### 2.8 Electronic senses analysis

Electronic senses were evaluated by Sheng et al. with some modification (Zheng et al., 2025). Electronic tongue (ASTREE I, Alpha. MOS, France) was composed of seven chemical sensors: AHS (Sourness), PKS (Synthesize 2), CTS (Saltiness), NMS (Umami), CPS (Synthesize 1), ANS (Sweetness), and SCS (Bitterness). Electronic nose (PEN3, AIR SENSE, German) sensor array including WlC (aromatic compounds), W5S (polar compounds, nitrogenoxides, ozone), W3C (ammonia, aldehydes, ketones), W6S (hydrogen), W5C (aromatic and aliphatic, alkanes), WlS (methane, broad-methane), W1W (sulphur compounds, terpenes), W2S (alcohols), W2W (sulphur organic compounds), and W3S (methane-aliphatic). Electrical resistivity was used to denote the sensor response.

### 2.9 Flavonols dissolution assessment

#### 2.9.1 Dissolution curves

Dissolution kinetics is a science used to study the rate and mechanism of drug release from solid systems into dissolution media. After crushing SB fruits in a juicer for 1–5 min respectively, timing was immediately initiated. Samples were collected at 0, 5, 10, 20, 30, 40, 50, 60, 120, and 180 min post-mechanical crushing, respectively, and centrifuged at 8000 rpm for 5 min. The supernatants which represent the dissolution amount were collected and extracted, and finally determined by HPLC. The dissolution curve of flavonols over time was plotted using time points and flavonol content as indicators.

#### 2.9.2 Dissolution rate

The dissolution rate is a key index in the evaluation of dissolution effect, which can directly reflect the dissolution of flavonols. The dissolution rate (%) was calculated by instantaneous dissolution content of flavonol relative to the flavonol content of sample.

### 2.10 Kinetics of dissolution

In order to clarify the dissolution behavior of flavonols from system, it is essential to choose appropriate kinetic models for fitting. According to dissolution mechanism of flavonols, the experimental data were substituted into First-order kinetic model (Eq. 5) and Weibull kinetic models (Eq. 6) for fitting, and the suitable models were selected according to the R^2^ value.

(5) $$y=a_{0}+{\left(a-a_{0}\right)}^{*}\;\left(1-\exp^{-\mathrm{kx}}\right)$$

(6) y=A0+A−A0∗1−exp−kx−xc^d

where, “a” and “A” were the maximum dissolution content; “a_0_” and “A_0_” were the initial dissolution content; “k” represented the dissolution rate constant; “xc” was the delayed dissolution term, which represents the lag time before dissolution begin; “d” was the shape parameter, which describes the shape of the dissolution curve.

### 2.11 Biological activity

#### 2.11.1 α-glucosidase inhibition capacity

The α-glucosidase inhibition rate was determined according to a study (Flores et al., 2013) with simple adjustments, and all experimental reactions were carried out in black 96-well plate with a transparent bottom. 50 μL sample or extract was mixed with 25 μL α-glucosidase (0.4 U/mL) and incubated at 37 °C for 10 min. The reaction was initiated by the addition of 25 μL 4-nitrophenyl β-D-glucopyranoside (PNPG) and incubated at 37 °C for 20 min. Finally, the reaction was terminated by the addition of 100 μL Na_2_CO_3_. Phosphate buffer and acarbose solution (0.005–0.3 μg/mL) were added to replace samples as the negative and positive control groups. The absorbance values of different reaction groups were measured at 400 nm, and the α-glucosidase inhibition results were calculated by the formula as follows. IC_50_ was the sample concentration when the α-glucosidase inhibition is 50 %.

(7) $$\alpha -\mathrm{glucosidase inhibitory}\;\left(\%\right)=1-\frac{{\mathrm{OD}}_{\text{sample}}}{{\mathrm{OD}}_{\text{control}}}\times 100$$

#### 2.11.2 ORAC

The operation was carried out in a 96-well plate with a black transparent bottom. First, 20 μL Trolox or sample extract of different concentrations were added, then 120 μL fluorescein solution (70 nM) was added under dark conditions, and finally 60 μL 2,2′-Azodiisobutyramidine dihydrochloride (AAPH) (12 mM) solution was added. After mixing, it was immediately put into the 37 °C microplate reader. The excitation and emission wavelengths were set as 485 nm and 520 nm, respectively. The fluorescence value was read every 1–2 min, and the change of fluorescence intensity with time was recorded until the fluorescence value dropped below 5 % of the initial value (Huang et al., 2002). According to the fluorescence attenuation curve formed by the fluorescence value at different time points, the area under the curve (AUC) was calculated, and the ORAC value of the sample was calculated by using the Eq. 8:

(8) $$\mathrm{ORAC}\;\left(\mathrm{\mu mol}\;\mathrm{TE}/\mathrm{mL}\right)=\left(\text{AUCsample}-\text{AUCCON}\right)/\left(\text{AUCTrolox}-\text{AUCCON}\right)\times C_{\text{Trolox}}\times F$$

Where, F is the dilution ratio of samples.

### 2.12 Storage stability

To evaluate the stability of the juice system and changes in flavonol contents under juicing-induced disruption, the juice samples were stored at 4 °C. Over a 28 d period, with assessments conducted per 7 d, the appearance of the samples was monitored. Additionally, the samples were centrifuged at 8000 rpm for 10 min to determine the total flavonoid content in the supernatant, as well as the contents of three specific flavonols (quercetin, kaempferol, and isorhamnetin). All experiments were performed in triplicate, and the results were expressed as the mean value.

### 2.13 Data analysis

All experiments and tests were repeated at least three times. Images related to the experiments were captured with a Canon digital camera and processed with Adobe illustrate CS6. The experimental data were statistically analyzed using IBM SPSS statistics 26. Plotted, bar chart, line chart, radar chart, PCA and clustering analysis, were employed to explore the differences in physical characters, flavonols dissolution effect and chemical sensory properties among SB juice by using Graphpad Prism 9 and Origin 2018. *p* < 0.05 was considered statistically significant.

## 3 Results and discussion

### 3.1 Physiochemical characteristics

As a food and drug homologous material exhibiting diverse biological activities, the physiochemical properties of SB constitute essential research elements. As shown in Fig. S1, SB was characterized by high acidity (pH 3.00) and 79.97 % water content. This acidic environment stabilized key antioxidants (e.g., vitamin C, flavonoids, polyphenols), but also slowed oxidative degradation, thereby prolonging the shelf life of both fresh berries and derived products. The measured pH of the sea buckthorn system was 3.0, confirming its inherent acidity. This acidic environment is likely attributable to the high content of organic acids, such as malic acid and quinic acid, within the fruit. This acidity may contribute to the stabilization of bioactive compounds present in sea buckthorn (Wang et al., 2022). The analyzed SB samples demonstrated favorable physicochemical properties (9.87°Brix soluble solids, 5.54 g CA/kg titratable acidity, and 2.40 g/100 g total sugars), meeting the quality standards required for subsequent experimental investigations.

### 3.2 Impact of crushing methods on TFC releasement

Fig. S1 revealed significantly reduced (*p* < 0.05) TFC levels in mechanically processed SB versus the intrinsic TFC level of untreated material (5.02 mg CE/mL). High-pressure homogenization achieved maximal TFC (2.64–3.58 mg CE/mL), outperforming juicing (2.31–3.12), shear homogenization (1.38–2.67), ultrasonication (2.16–3.01), colloid milling (1.94–3.07) mg CE/mL. It is likely resulted from its dual-phase disruption mechanism: hydrodynamic shear forces under high pressure (typically 50–110 MPa) and impact forces from grinding beads. This combined action generated more extensive cell wall fracture than single-mechanism methods, facilitating efficient phytochemical transfer to the solvent phase (Ahmed & Eun, 2018). The TFC remained pressure-insensitive (*p* > 0.05 across 50–110 MPa), may due to counteracting effects that enhanced extraction and concurrent polyphenol oxidase (PPO)-mediated degradation for phenolics in apple, grape and orange juice (He et al., 2016). Notably, jujube pulp retained TFC even at 400 MPa (Shen et al., 2016) whereas lemon juice exhibited losses from quercetin activation (Du, 2016), highlighting species-dependent pressure responses. While other treatments showed comparable TFC (p > 0.05), juicing exhibited time-dependent variation (peak at 4 min, decline by 5 min). Shearing processing yielded significantly lower TFC (*p* < 0.05), likely due to combined cellular disruption-induced oxidase release and thermal degradation. The duration-dependent TFC enhancement (8–17 % increase from 10 to 120 min) align with findings reported by Wang et al., 2019. Ultrasonic cavitation effects generate localized microjets and shockwaves capable of inducing microstructural modification, selective permeabilization of cell membranes and liberated intracellular flavonoids. Colloid mill processing exhibited time-dependent TFC extraction efficiency, peaking at 40 min (3.07 mg CE/mL) before decline, may due to combined thermal and mechanical degradation effects during extended operation. Both industrial and household methods achieved comparable TFC extraction. From a consumer perspective, household processing may be more suitable for daily dietary applications. Hydrophobic flavonol release efficiency remains a critical issue warranting further investigation.

### 3.3 Flavonol dissolution content

The results indicate no statistically significant difference in TFC across the tested mechanical crushing methods. SBF consist primarily of hydrophilic compounds, with a minor proportion of hydrophobic constituents. This suggested that mechanical crushing methods may not substantially impact the release of hydrophilic flavonoids. Conversely, hydrophobic compounds are predominantly present in bound forms within the pulp matrix. Given their notable bioactive properties, research focus was directed toward investigating the release efficiency of hydrophobic flavonols. The concentration of major SBF was 180.37 μg/g, 124.55 μg/g and 146.59 μg/g, respectively, which was consistent with previous studies (Table S1) (Guo et al., 2017). Three compounds exhibited distinct temporal release patterns during mechanical processing. Quercetin showed time-dependent accumulation (2.25 μg/g at 5 min) through β-glucosidase mediated deglycosylation of rutin analogues. However, the dissolution rate was 1.24 % compared with the total quercetin. HPLC analysis revealed time-dependent kaempferol: undetectable at *t* < 2 min, peaking at 3 min (1.75 μg/g), then decreasing to 0.01 μg/g at *t* = 5 min). This suggested initial energy induced liberation from hydrophobic binding sites precedes compound instability in the aqueous phase. The raw juice extraction process released isorhamnetin at concentrations of 0.33–0.63 μg/g, representing only 0.23–0.43 % of the total available content. This low extraction efficiency suggests strong matrix retention effects, potentially due to isorhamnetin's structural interactions with cell wall components or limited solubility under the processing conditions. Comparative analysis revealed SBF dissolution efficiency order was quercetin> isorhamnetin> kaempferol. The time-dependence of quercetin release versus the temporal stability of other flavonols addresses the need for targeted extraction strategies for hydrophobic phytochemicals. Previous studies demonstrate that quantitative structure-property relationship (QSPR) analyses identify flavonoid conformations with torsion angles approximating 40° as exhibiting optimal solubility. This establishes a definitive correlation between molecular conformation and dissolution behavior in aqueous systems (Chebil et al., 2007). Flavonols (mean θ = 12.3°) showed 3.2 fold lower aqueous solubility than flavanones (θ = 28.7°), with intramolecular H-bonding accounting for 41 % of variability within the flavonol subgroup (Dong et al., 2023).

### 3.4 Fruit pulp characteristics

**Fig. 1:**
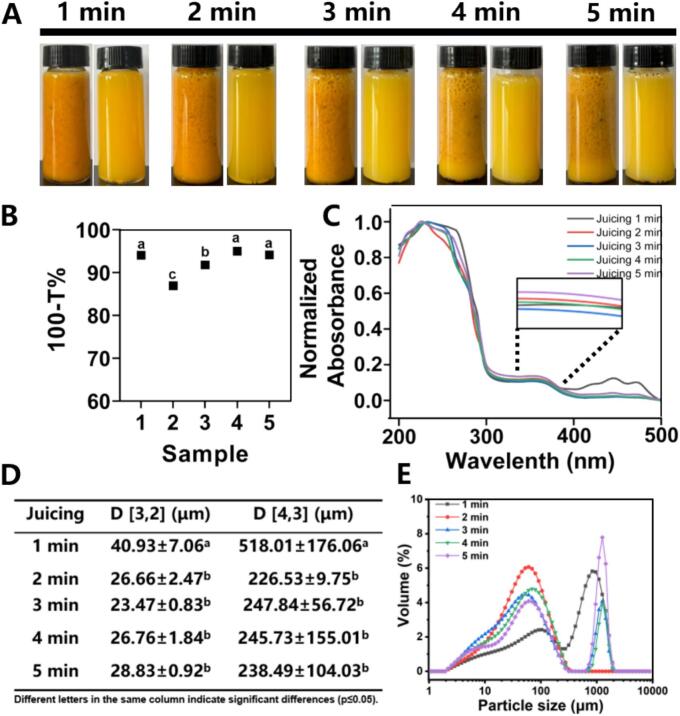
The macroscopic morphology of sea buckthorn juice before and after centrifuge (A), turbidity (B), UV–visible wavelength scanning (C), and pomace particle size of SB system (D, E). Different letters in the same column indicate significant differences (*p* < 0.05).

Macroscopic homogeneity, turbidity, chroma, particle size distribution and microstructure were employed to comprehensively evaluate system stability and pulp fragmentation degree. As illustrated in Fig. 1A, prolonged crushing duration induced a progressive lightening of juice coloration, with the pulp transitioning from orange to bright yellow. Chromatic results showed that there were significant differences in the L* values among all samples (ranging from 46.37 to 52.94) (Table S2). As the juicing duration increased from 1 to 4 min, the lightness of the samples increased, followed by a decrease when juicing for 5 min. The a* values (14.12–20.29) and b* values (40.24–49.04) of the samples gradually decreased with the extension of juicing time, indicating that juicing and crushing would reduce the red and yellow saturation of the samples. This changing trend is consistent with the appearance color of the samples. Mechanical crushing transforms the original orange-yellow color of SB into a pale yellow, thereby reducing the red and yellow values of the samples. In addition, the ΔE value also showed a downward trend with juicing time (43.47–46.33), indicating that the color variation of the juice under different juicing durations decreased, and the color gradually became consistent, which suggests an improvement in the stability of the samples (Wang et al., 2025). This chromatic shift can be attributed to the differential stability and distribution of key pigments carotenoid and flavonoid in SB (Gherasim et al., 2024). Carotenoid is the mainly contributor to orange pigmentation and the mechanical fragmentation enhances oxidative susceptibility via increased air interface exposure and formation of epoxy/ketone derivatives (Maoka, 2020). While flavonoids dominant pigments for light yellow in juice and enhanced aqueous dispersion due to cellular disruption and pericarp-derived soluble fiber complexation. Prolonged crushing (> 4 min) reduced pulp particle density, inducing flotation, aggregation, and system stratification, ultimately decreasing turbidity (Fig. 1B). It has been demonstrated that juice turbidity is primarily attributed to the interaction between turbidifying active proteins and polyphenols, which form insoluble multi-molecular complexes (Jiménez-Sánchez et al., 2017). Pulp particle size distribution spanned 1–2000 μm (Fig. 1E), with mean particle diameter decreasing significantly (*p* < 0.05) from 40.93 μm to 23.47 μm after 1 min crushing (Fig. 1D), demonstrating time-dependent fragmentation efficiency.

The solubility of flavonols was investigated by UV–Vis scanning. Fig. 1C illustrated the UV scanning spectra of the extraction of SBD at different fragmentation times. The flavonols characteristic absorption peaks at 370 nm were focused for excluding the same absorption range of other compounds like rutin at 260 nm. UV–Vis spectra (0.1–0.2) confirmed SBF liberation, consistent with established phytochemical-optical relationships: high flavonol content typically correlates with low UV reflectance (Dudek et al., 2020). The findings indicate that the absorption spectrum of SBF exhibits a concentration-dependent characteristic, whereby a higher flavonol content corresponds to a lower ultraviolet reflectance.

**Fig. 2:**
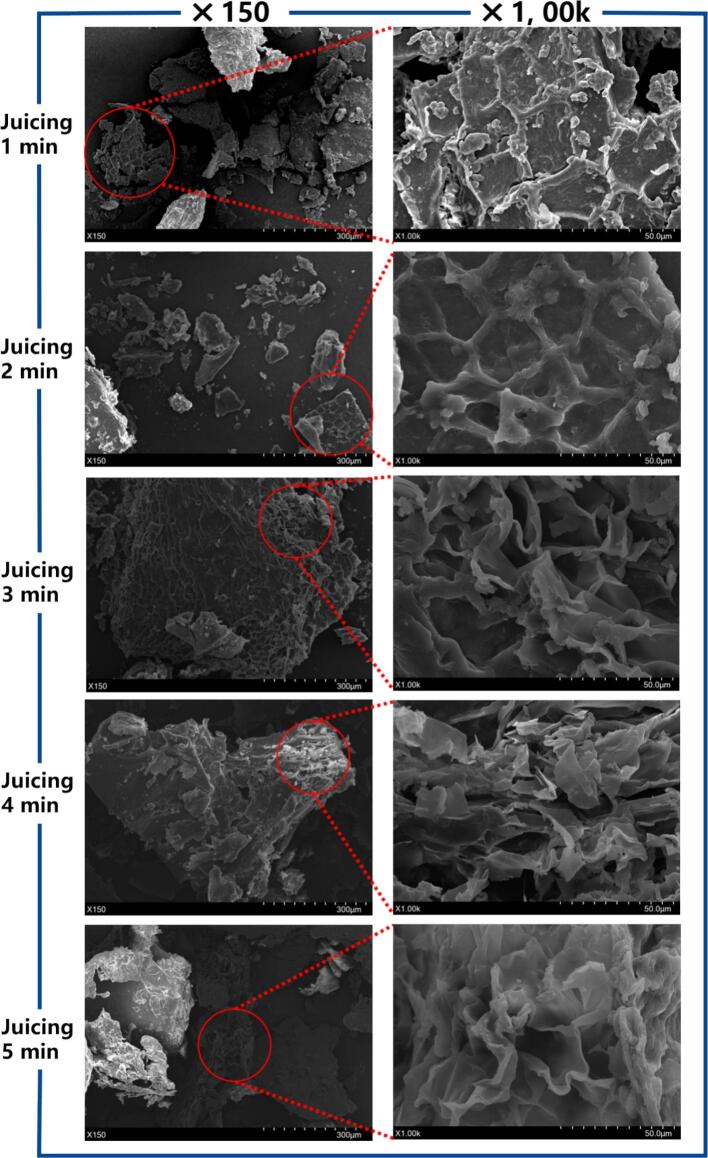
The SEM morphology of SB pomace after juicing 1, 2, 3, 4, 5 min.

To observe the variations in the tissue of sea buckthorn pomace at different juicing durations, the micro-morphology of the samples was inspected using SEM at magnifications of 150 x and 1000 x (Fig. 2). The pomace fragments exhibited an irregular shape, which might be attributed to the crushing mode of the cutter head and the possible existence of uneven crushing. When the crushing duration was less than 2 min, the reticular structure on the surface of the pomace was uniformly distributed without obvious damage traces. However, when the juicing duration exceeded 3 min, the reticular structure of the pomace presented obvious rupture, with variable pore sizes and disordered arrangement. The sample juiced for 4 min demonstrated the most significant degree of damage. Mechanical force caused damage to the structural substances such as cellulose and lignin in the pomace, resulting in their fracture and thereby presenting a disorderly microstructure. This result was similar to the previous research findings on the ordered-to-disordered change in the microstructure of mulberry pulp under different juicing conditions (Li et al., 2016). The changes in the microstructure of the pomace exhibited a similar trend to those in its particle size distribution, indicating that different juicing durations indeed disrupted the pomace tissue.

### 3.5 Assessment of electronic senses

MOS electronic sensory sensors assessed SB juice from varying juicing durations. *E*-tongue results revealing that juicing mechanical crushing affected taste differently: juicing 5 min showed stronger bitterness, sweetness, saltiness but weaker sourness and umami. In contrast, juicing 3 min and 4 min samples had slightly higher sourness and umami, which might be attributed to the release of SB juice characteristic flavor compounds induced by juicing mechanical crushing (Tkacz et al., 2020) (Fig. S2A). PCA result (99.6 % total variance, PC1:98.0 %, PC2:1.6 %) showed juicing 1–4 min groups on positive semi-axis and the juicing 5 min group on negative semi-axis of PC1, indicating 5 min juicing crushing impacts sample taste (Fig. S2B). However, e-nose responses and PCA distribution (94.7 % total variance, PC1:80.9 %, PC2:13.8 %) revealed no significant flavor differences, with samples clustered together (Fig. S2C, S2D). Overall, juicing-related mechanical crushing affected taste but not flavor, aiding consumers in choosing juicing durations per preferences during home preparation.

### 3.6 Flavonol dissolution evaluation

#### 3.6.1 Macroscopic morphology and UV–Vis scanning

**Fig. 3:**
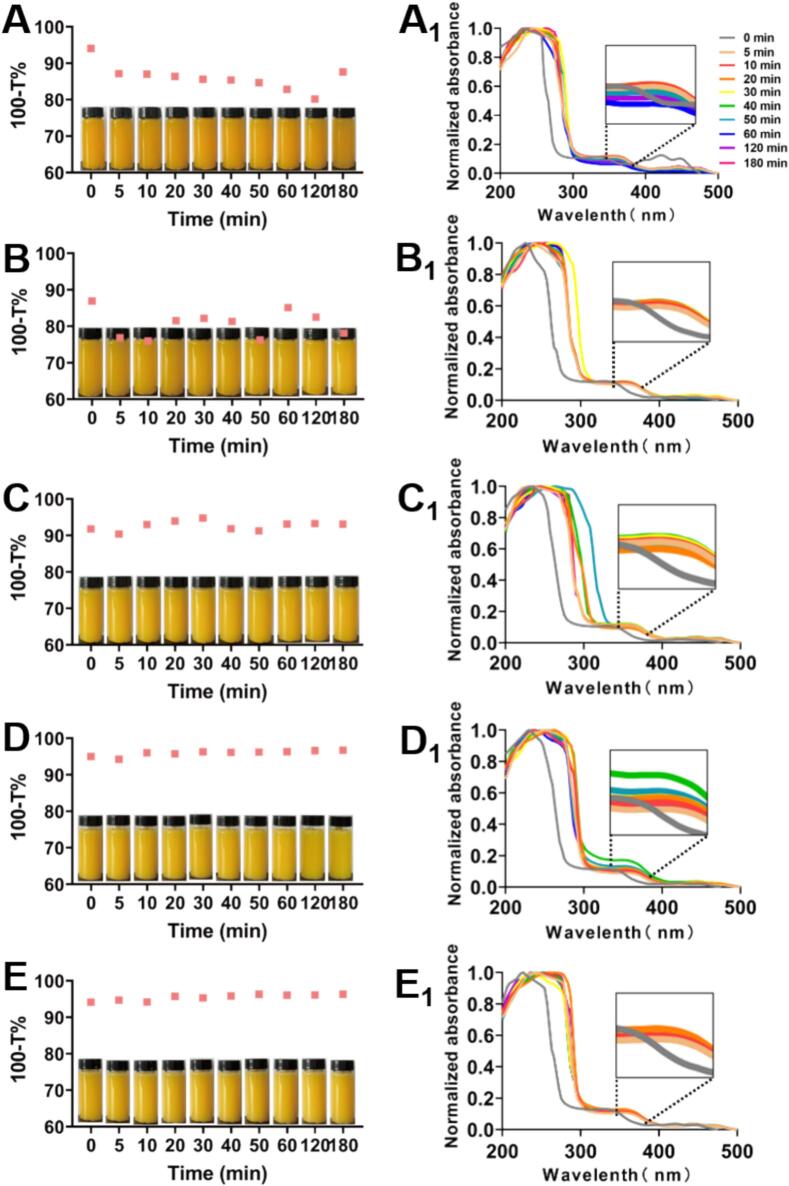
The turbidity (A-E) and UV–visible wavelength scanning (A_1_-E_1_) of SBD at 0, 5, 10, 20, 30, 40, 50, 60, 120, and 180 min after juicing 1–5 min. A&A_1_: Juicing 1 min；B&B_1_: Juicing 2 min；C&C_1_: Juicing 3 min；D&D_1_: Juicing 4 min；E&E_1_: Juicing 5 min.

The physical morphology and release behavior of SBF from cell structure under juicing are of great significance for household process in this field. The fruit juice obtained by centrifuging sea buckthorn pulp at different time points (0, 5, 10, 20, 30, 40, 50, 60, 120, 180 min) exhibits a bright yellow color, uniform texture, and no significant precipitation. Turbidity analysis revealed time-dependent stabilization patterns. Shorter crushing (1–2 min) allowed sedimentation of larger particles and exhibited lower turbidity, whereas prolonged crushing (3–5 min) produced stable colloids and constant turbidity (Fig. 3A-E). This size-dependent behavior aligned with Stoke's law predictions for particulate suspension (Tarafdar & Kaur, 2021).

The dissolution behavior of flavonols were characterized by UV–Vis spectrophotometry (200–500 nm; Fig. 3 A_1_-E_1_). Distinct absorption maxima suggested the predominant release of soluble phytochemicals, including vitamin C, catechins, and flavonol glycosides. Characteristic flavonol absorbance (300–400 nm) exhibited time-dependent spectral shifts. The 2 min juicing group showed maximal absorption at 370 nm after 40 min fragmentation. The 3 min group demonstrated a bathochromic shift at 50 min, potentially indicating glycosyltransferase-mediated flavonoid modification. To validate these observations, targeted HPLC-DAD analysis was employed for compound-specific quantification and structural identification.

#### 3.6.2 TFC and dissolution curve of SBF

**Fig. 4:**
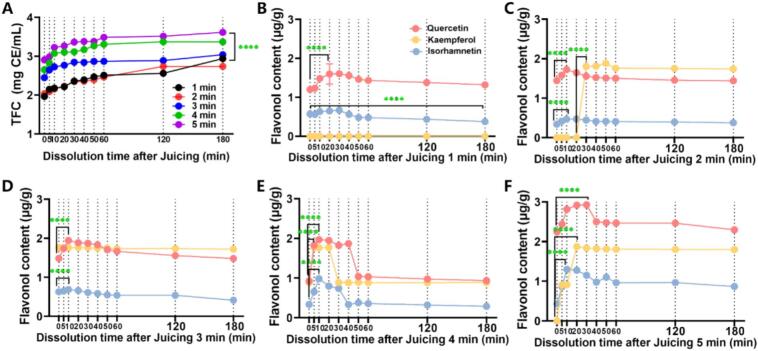
The TFC (A) and SBF content (quercetin, kaempferol, and isorhamnetin) (B—F) of SBD at 0, 5, 10, 20, 30, 40, 50, 60, 120, and 180 min after juicing 1–5 min. B: Juicing 1 min；C: Juicing 2 min；D: Juicing 3 min；E: Juicing 4 min；F: Juicing 5 min. Graphs represented mean ± SD from three independent experiments (p < 0.05). Values and error bars represent the means and standard deviations of duplicated experiments (*n* = 3), respectively. The *P* values were computed by two-tailed Student's *t-*test (*p < 0.05, **p < 0.01, ***p < 0.001, ****p < 0.0001, ns represents no significance).

The released TFC and SBF were further monitored at 0, 5, 10, 20, 30, 40, 50, 60, 120 and 180 min following mechanical crushing. As shown in Fig. 4A, TFC accumulation exhibited time-dependent enhancement, with 5 min juicing yielding significantly higher levels than 1 min juicing, consistent with reports that reamer methods improve methoxy-flavonoids dissolution through more effective mechanical disruption (Li et al., 2021). This trend may be further augmented by SB lipids, as their concurrent release could promote flavonoid solubilization via oil-mediated dissolution.

The contents of quercetin, kaempferol, and isorhamnetin (three flavonols) were determined by HPLC at different time points after different juicing durations (Fig. 4B-4F). The dissolution ranges of quercetin, kaempferol and isorhamnetin were 1.20–2.92 μg/g, 0–1.88 μg/g, 0.56–1.29 μg/g, respectively. The dissolution content of the three substances increased rapidly at 0–20 min and then gradually decreased and tended to be stable. Among the flavonols detected in the SBD, quercetin was the most abundant. The contents of kaempferol and isorhamnetin exhibited distinct trends depending on juicing duration. Notably, kaempferol was undetectable after 1 min of extraction but appeared at higher levels than isorhamnetin when juicing exceeded 2 min. Comparative analysis revealed significantly lower endogenous levels of kaempferol in SB relative to the other flavonols. This inherent scarcity resulted in sub-threshold concentrations during initial juicing phases, precluding analytical detection. Besides, The polarity and solubility of flavonols are primarily determined by their hydroxyl (-OH) groups. Structurally, the B-ring of quercetin contains two phenolic hydroxyl groups, whereas kaempferol has only one. In contrast, isorhamnetin bears one hydroxyl and one methoxy (-OCH₃) group, the latter being less polar than a hydroxyl group. Therefore, under extended extraction conditions, kaempferol demonstrated greater extractability than isorhamnetin, despite its lower native abundance.

#### 3.6.3 SBF dissolution rate

**Fig. 5:**
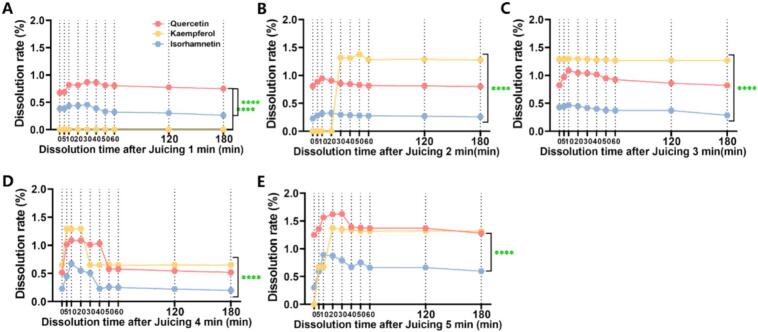
The dissolution rate of SBF at 0, 5, 10, 20, 30, 40, 50, 60, 120, and 180 min after juicing 1–5 min. A: Juicing 1 min；B: Juicing 2 min；C: Juicing 3 min；D: Juicing 4 min；E: Juicing 5 min. Graphs represented mean ± SD from three independent experiments. Values and error bars represent the means and standard deviations of duplicated experiments (n = 3), respectively. The P values were computed by two-tailed Student's t-test (*p < 0.05, **p < 0.01, ***p < 0.001, ****p < 0.0001, ns represents no significance).

The instantaneous dissolution rate quantifies the time-dependent release flux of bioactive compounds from the solid matrix, providing mechanistic insights into the dynamic dissolution behavior. Following mechanical crushing (180 min), quercetin, kaempferol, and isorhamnetin exhibited dissolution rates of 0–2 % (Fig. 5). Temporal analysis revealed distinct kinetic profiles that quercetin and isorhamnetin displayed unimodal release patterns (initial increase followed by stabilization) while kaempferol was undetectable (<LOD) at 1 min. Notably, dissolution kinetics at 2, 4, and 5 min followed similar trends, whereas the 3 min group demonstrated near-constant release rates. These observations suggest juicing duration modulates flavonol release kinetics, with optimal temporal stability observed at 3 min. The biphasic mechanism combines initial diffusion-controlled release (*t* < 3 min) with subsequent dissolution-dominated kinetics (*t* ≥ 3 min).

### 3.7 Flavonols dissolution kinetics

Current research on flavonoids predominantly emphasizes industrial processing methodologies (Lan et al., 2023), physiological activities and bioavailability (Teng et al., 2023). However, these investigations have primarily examined purified flavonoids, while largely neglecting the dissolution kinetics of dietary flavonols during household food processing. This knowledge gap underscored the need to elucidate the relationship between household mechanical processing and flavonols release. To address this, we employed first-order kinetic and Weibull models to characterize the temporal dissolution profile of SBF. These models were fitted to experimental data obtained at various time points following mechanical juicing, with SBF dissolution content as the quantitative indicator. This approach enabled systematic investigation of flavonol dissolution behavior under mechanical fracturing and time-dependent dissolution kinetics, while accounting for raw material properties and comminution methods (Jurinjak Tušek et al., 2022).

**Fig. 6:**
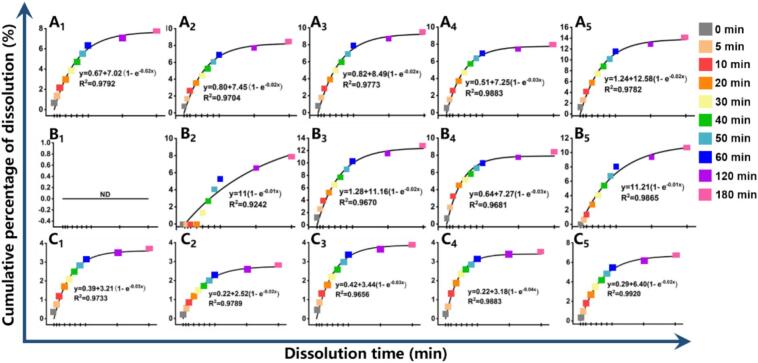
The dissolution kinetic curves of quercetin (A_1_-A_5_), kaempferol (B_1_-B_5_), and isorhamnetin (C_1_-C_5_) at 0, 5, 10, 20, 30, 40, 50, 60, 120, and 180 min after juicing 1–5 min fitting by First-order model. A_1_&B_1_&C_1_: Juicing 1 min；A_2_&B_2_&C_2_: Juicing 2 min；A_3_&B_3_&C_3_: Juicing 3 min；A_4_&B_4_&C_4_: Juicing 4 min；A_5_&B_5_&C_5_: Juicing 5 min.

**Fig. 7:**
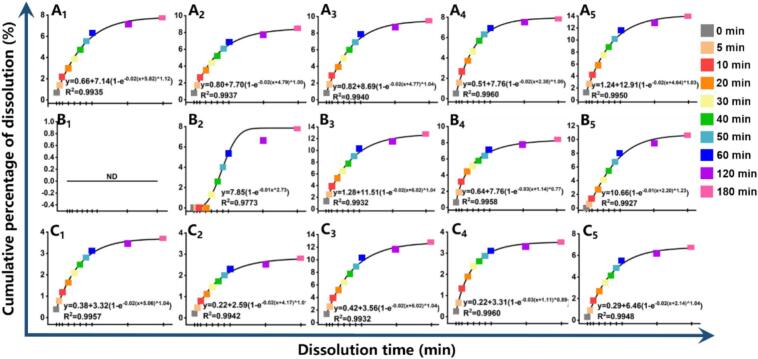
The dissolution kinetic curves of quercetin (A_1_-A_5_), kaempferol (B_1_-B_5_), and isorhamnetin (C_1_-C_5_) at 0, 5, 10, 20, 30, 40, 50, 60, 120, and 180 min after juicing 1–5 min fitting by Weibull model. A_1_&B_1_&C_1_: Juicing 1 min；A_2_&B_2_&C_2_: Juicing 2 min；A_3_&B_3_&C_3_: Juicing 3 min；A_4_&B_4_&C_4_: Juicing 4 min；A_5_&B_5_&C_5_: Juicing 5 min.

Quercetin exhibited a dissolution rate constant (k) of 0.02 s-1 during initial juicing (<3–5 min), peaking at 0.03 s-1 at 4 min (Fig. 6 A_1_-A_5_). This pattern was confirmed by Weibull modeling, which produced an identical baseline k value of 0.02 s-1 (Fig. 7 A_1_-A_5_; Table S3). Kaempferol exhibited dissolution rate constants (k) ranging from 0.01 to 0.03 s-1 in both models, demonstrating no significant temporal dependence (Fig. 6 B_1_—B_5_, Fig. 7 B_1_—B_5_)) (*p* > 0.05). The k of isorhamnetin was slightly higher in the first-order kinetic model compare to the Weibull model (Fig. 6 C_1_—C_5_, Fig. 7 C_1_—C_5_). Insufficient crushing time (<2 min) limited compound diffusion from the plant matrix into the solvent, while prolonged processing induced thermal degradation of bioactive components due to mechanical heat generation, consequently reducing flavonol dissolution rates (Benítez-Correa et al., 2025). The differential dissolution kinetics observed among flavonols suggest tissue-specific binding affinities or molecular interactions within plant cellular structures. The shape parameter (d) in the Weibull model characterizes the dissolution profile: Decelerating dissolution rate (d < 1); First-order kinetics (exponential decay) (d = 1); Accelerating dissolution rate (d > 1) (Woloszyn et al., 2022). Quercetin dissolution exhibited d ≥ 1 values at all juicing durations except 1 min (d < 1), confirming first-order kinetic behavior (d = 1) and time-enhanced dissolution (d > 1) under most processing conditions (Table S3). With the exception of the 4 min group, both kaempferol and isorhamnetin exhibited d > 1, indicating progressively increasing dissolution rates over time. This trend was particularly pronounced in the 2 min group. In contrast, the 4 min group demonstrated d < 1, suggesting time-dependent deceleration of dissolution kinetics. This anomalous behavior may be attributed to limited mechanical disruption efficacy for liberating bound flavonoid forms, and potential thermal degradation induced by prolonged blade rotation. Within 180 min post-crushing, three flavonols exhibited distinct release profiles: quercetin showed highest extractability (14.15 %), followed by kaempferol (12.79 %) and isorhamnetin (6.75 %). Quantitative evaluation revealed high correlation coefficients (R^2^ > 0.95) for flavonol dissolution fitting, excepting kaempferol at 2 min. The Weibull model (R^2^ > 0.97) significantly outperformed first-order kinetics (*p* < 0.05). Weibull parameter analysis (d > 1) supports a matrix degradation-controlled release mechanism over Fickian diffusion (Martín-Camacho et al., 2023). The diffusion and mass transfer of solvents and solutes are predominant factors in the extraction process (Zhou et al., 2023). The release of flavonoid compounds from plant tissues involves a multi-stage diffusion process, comprising: Intracellular diffusion of free flavonoid glycosides across the tonoplast into the cytosol, and extracellular diffusion of bound aglycones through the pectin-cellulose network followed by plasma membrane efflux (Fig. S3) (Yang et al., 2019). Mechanical fragmentation accelerates solvent penetration, and subsequent pectin hydrolysis in acidic medium (pH 2–4) drives flavonol dissolution (Fig. S3). The dense, low-porosity structure of SB restricts diffusion, rendering matrix dissolution the dominant release pathway. Although flavonols are stable at pH 3–4, their poor solubility in stronger acids may compromise extraction efficiency (Shen et al., 2023).

### 3.8 Functional activity

**Fig. 8:**
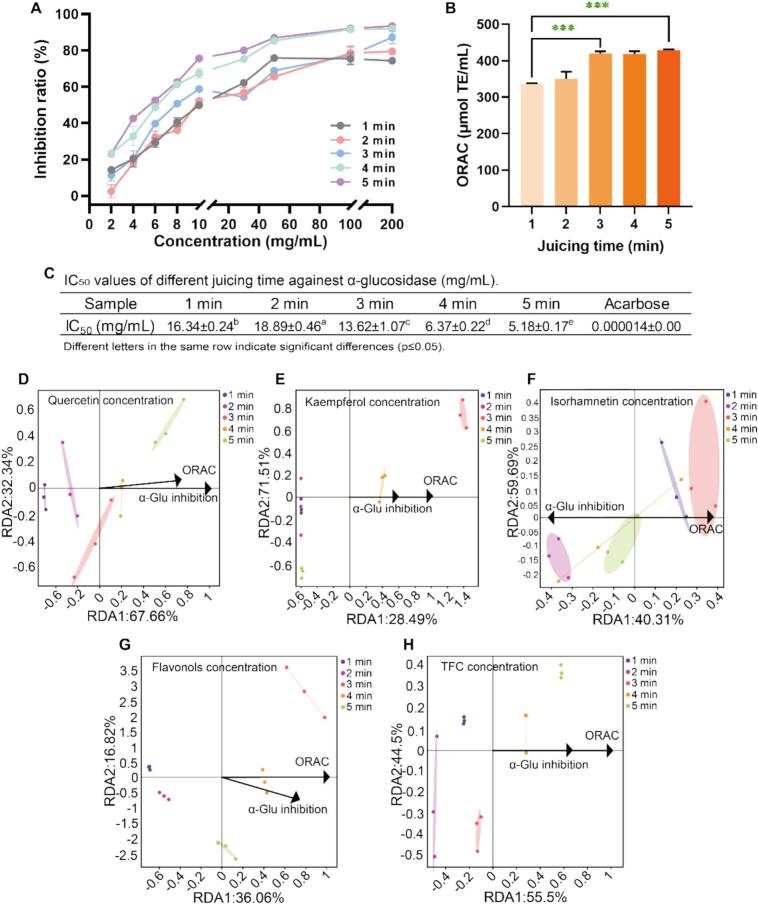
The α-glucosidase inhibition rate and ORAC effects of SBD after juicing 1–5 min (A-C). The PCA between quercetin, kaempferol, isorhamnetin, total flavonols, TFC and juice bioactivities, respectively (D-H). Different letters in the same row indicate significant differences (p < 0.05). Values and error bars represent the means and standard deviations of duplicated experiments (n = 3), respectively. The P values were computed by two-tailed Student's t-test (*p < 0.05, **p < 0.01, ***p < 0.001, ****p < 0.0001, ns represents no significance).

Previous studies confirmed that flavonoids, particularly SB-derived hydrophobic flavonols, mediated antioxidant and anti-α-glucosidase activities (Gong et al., 2020; Ma et al., 2022). The dissolution efficacy of flavonols during mechanical fragmentation was evaluated by analyzing the biological activity of SBD. As shown in Fig. 8, the extracts exhibited significant α-glucosidase inhibitory activity (indicating carbohydrate digestion modulation) and ORAC. The α-glucosidase inhibitory activity of SB extracts showed as IC_50_. All samples displayed the effective inhibition on α-glucosidase with SB concentration IC_50_ range from 5.18 to 18.89 mg/mL (Fig. 8A). α-glucosidase inhibition of samples was followed a time-dependent pattern, correlating with TFC. Studies have confirmed that the fruit and vegetable-originated compounds have great α-glucosidase inhibition effect (Bie et al., 2024). Certain phenolic compounds affect the polypeptide chains elongation through bind to α-glucosidase. This leads to changes in the microenvironment around tryptophan or tyrosine residues in α-glucosidase, as well as rearrangement of the secondary structure, ultimately resulting in enzyme inactivation (Qin et al., 2023; Yang et al., 2023). This suggested flavonoid-dependent inhibition, consistent with reported synergistic effects between luteolin and quercetin in hypoglycemic food development (Pan et al., 2025).

The ORAC assay serves as a biologically significant indicator of antioxidant capacity, with demonstrated physiological relevance (Tkacz et al., 2021). ORAC values (Fig. 8B) increased with juicing time (max at 5 min), reflecting time-dependent release of antioxidants. Above all, the PCA of SBF and juice bioactivity showed positive correlations between the α-glucosidase inhibition rate, ORAC values of samples and quercetin/total flavonol/TFC concentration at juicing 3–5 min, kaempferol at juicing 3–4 min (Fig. 8D-H). Conversely, isorhamnetin concentration generally exhibited negative correlations with both bioactivity indices. Specifically, isorhamnetin correlations were inconsistent: positive correlations were observed specifically at juicing 2, 5 min for α-glucosidase inhibition, and at juicing 1, 3 min for ORAC. These results demonstrated that while total flavonol/flavonoid concentration correlates with juice bioactivity, the relationship for isorhamnetin is complex and time-dependent. This suggests differential impacts of released flavonoid concentration by juicing on bioactivity. However, other juice components (e.g., phenolics, organic acids, carotenoids) may also contribute significantly (Wang et al., 2022). Subsequent studies should comprehensively investigate the correlation strength and potential interactions of all relevant components with bioactivity.

### 3.9 Storage stability

Sample was stored at 4 °C for 28 d to further evaluate changes in the juice appearance and flavonols content (Fig. S2). The color of sample did not change significantly within 21 d; however, obvious stratification occurred by the 28 d. Larger particles (e.g. pulp, cellulose) gradually settled to the bottom, indicating that juicing-induced disruption failed to disperse the pulp tissue into uniform particles. This phenomenon affected the sample appearance and compromised the uniformity and integrity of its texture (Fig. S2E). Additionally, the TFC of juice rising from 2.86 mg CE/mL to 4.15 mg CE/mL, suggesting that total flavonoids were continuously released during storage process, thereby facilitating the stability of flavonoids and reducing their oxidative degradation (Fig. S2E). this might be due to that most of SBF are hydrophilicity glycosides, and they are more easily released from pulp tissue.

Despite the strong hydrophobicity of flavonoid aglycones, their beneficial bioactivities for human health prompted us to focus on changes in flavonol contents during storage. Quercetin exhibited the highest dissolution content in the supernatant (from 1.80 to 4.03 μg/g) (*p* < 0.05) after 21 d. While kaempferol did not change significantly and the highest content (2.02 μg/g) detected in the supernatant on 21 d (*p* > 0.05). Isorhamnetin showed a gradual dissolution trend during storage, and the highest concentration (2.58 μg/g) was detected on 28 d (p < 0.05) (Fig. S2F). Different dissolution degrees of flavonols may be related to the physical state of samples and the adsorption of flavonols by pulp particles, cell debris, fibers, pectin, and other substances (Fernandes et al., 2023). Some flavonols bind to particles, leading to their slow release and eventual sedimentation over time. While the low acidity of the system enhances the chemical stability of flavonols, it may also affect their solubility and degradation during long-term storage. The dissolution trends of flavonols may be associated with their different structure. The catechol structure of quercetin endows it with strong antioxidant activity but also makes it more susceptible to oxidation (Guo et al., 2022). Kaempferol is more stable due to its lack of catechol structure (Bangar et al., 2023), while isorhamnetin, as a 3-*O*-methylated derivative of quercetin, exhibits different degradation patterns compared to quercetin (Biswas et al., 2024). Furthermore, the weak enzymatic activity could catalyze the hydrolysis of flavonoid glycosides, as well as oxidative polymerization or degradation, ultimately influencing flavonol concentrations. These aspects explained the flavonols dissolution trend over time.

Besides, Considering the overall quality characteristics of juice, it is necessary to understand the quality characteristics of related products in the market. Huiyuan brand sea buckthorn 100 % NFC juice reported that the soluble solid content is 10°Brix, which is consist with the national industry standard GB/T 31121–2014 for fruit and vegetable juices and beverages. While the soluble solid content of our sample was 9.87°Brix. Samples exhibited higher TFC than Jixianfeng brand sea buckthorn juice (0.46 % in 500 mL). Furthermore, sample can be stored at 4 °C for 21 d while maintaining a good appearance, no separation, and stable flavonoid content. However, commercial sea buckthorn juice always has 2–12 months shelf life. This is related to the complex and frequent formulation adjustments of commercial products. Finally, we note that commercial products frequently undergo thermal stabilization or formulation adjustments (e.g., blending, pasteurization) that may differ from the non-thermal, small-scale mechanical crushing in our study. This distinction highlights that home juicing, while simpler, retains a bioactive profile that could offer a viable alternative for consumers seeking minimally processed options, even without direct functional activity comparisons. Future studies could include targeted analyses of commercial products using the same analytical framework to enable more robust comparisons.

## 4 Conclusion

Compared to shearing machines, ultrasonic devices, colloid mills, and HPP, home juicers exhibit no significant difference in the dissolution efficiency of TFC. However, significant differences (*p* < 0.05) were observed in the color and physical properties of the juice obtained by different juicing durations, with TFC ranging from 2.31 to 3.12 mg/mL. Flavonol content increased within 20 min after juicing, achieving 6 % cumulative dissolution rate. Flavonols dissolution behavior followed the diffusion-erosion composite mechanism (Weibull model, R^2^ > 0.97). The great α-glucosidase inhibitory capacity (IC_50_ was 5.18 mg/mL of SB) and ORAC (428.76 μmol TE/mL (p < 0.05) of SBD further confirmed the effective release of bioactives. These findings are attributed to the progressive mechanical disruption of the matrix, which modulates flavonoid release without altering the overall TFC dissolution efficiency. Juicing technology presents distinct advantages over NTP (HPP or ultrasonic treatment), including lower cost, operational simplicity, elimination of specialized training, and suitability for on-demand processing. These characteristics align well with the growing demand of non-thermal home food processing technologies. While the lower physical disruption of juicing may potentially limit compound release (although no impact on TFC was observed in this study), these limitations are offset by its usability for home applications prioritizing simplicity and cost-effectiveness. Furthermore, current research predominantly focuses on aglycones, neglecting flavonoid glycosides and other bioactive constituents. Future investigations should explore how processing parameters influence the co-release and stability of these multi-component bioactive substances, as their synergy is crucial for understanding the comprehensive nutritional value of SB.

## CRediT authorship contribution statement

**Jie Sheng:** Writing – review & editing, Writing – original draft, Investigation, Formal analysis, Data curation, Conceptualization. **Lanlan Yao:** Writing – original draft, Investigation, Formal analysis, Data curation, Conceptualization. **Qingying Dong:** Investigation, Data curation. **Bin Zhou:** Formal analysis, Conceptualization. **Bin Li:** Investigation, Data curation. **Guoqiang Zhang:** Writing – review & editing, Project administration, Conceptualization. **Hongshan Liang:** Writing – review & editing, Supervision, Project administration, Funding acquisition, Conceptualization.

## Declaration of competing interest

The authors declare that they have no known competing financial interests or personal relationships that could have appeared to influence the work reported in this paper.

## Data Availability

The authors do not have permission to share data.

## Appendix A Supplementary data

Supplementary material Image 1
