# Supplementary material for: Household non-thermal processing for the dissolution of sea buckthorn flavonoids and the effects on its bioactive characteristics
Source: Food Chem X. 2025 Aug 21;30:102941. doi: 10.1016/j.fochx.2025.102941 (PMC12491737; doi:10.1016/j.fochx.2025.102941)
Supplement: Supplementary file 1 — Supplementary material [file mmc1.docx]

**Household non-thermal processing for the dissolution of sea buckthorn flavonoids and the effects on its bioactive characteristics**

Jie Sheng ^a, d, 1^, Lanlan Yao ^a, d, 1^, Qingying Dong ^b^, Bin Zhou ^c^, Bin Li ^a, d^, Guoqiang Zhang^*^ ^b^, Hongshan Liang^* a, c^

^a^ College of Food Science and Technology, Huazhong Agricultural University, Wuhan, Hubei 430070, PR China

^b^ Wuhu Green Food Industry Research Institute Co., Ltd., Wuhu, 241000, China

^c^ Key Laboratory of Fermentation Engineering, Ministry of Education; National “111” Center for Cellular Regulation and Molecular Pharmaceutics; Hubei Key Laboratory of Industrial Microbiology; School of Biological Engineering and Food, Hubei University of Technology, Wuhan 430068, China

^d^ Key Laboratory of Environment Correlative Dietology (Huazhong Agricultural University), Ministry of Education, Wuhan, Hubei 430070, PR China

1: These authors equally contributed to this study.

*Corresponding author: Guoqiang Zhang, Hongshan Liang

E-mail address: [zhangguoqiang@ahpu.edu.cn](mailto:zhangguoqiang@ahpu.edu.cn)；[lianghongshan@mail.hzau.edu.cn](mailto:lianghongshan@mail.hzau.edu.cn)

**Figure captions:**

**Fig. S1.** The physiochemical indexes and TFC of SB treated by five non-thermal processing techniques. All data were presented as means and standard derivations. The results were statistically significant (p < 0.05). Values and error bars represent the means and standard deviations of duplicated experiments (n=3), respectively. The P values were computed by two-tailed Student’s t test (*p < 0.05, **p < 0.01, ***p < 0.001, ****p < 0.0001, ns represents no significance).

**Fig. S2.** Assessment of electronic senses and storage evaluation in juices. A&B: E-tongue data radar chart and PCA analysis of samples at different juicing time. C&D: E-nose data radar chart and PCA analysis of samples at different juicing time. E: Macroscopic morphology and TFC during 28 d storage. F: Supernatant concentrations of quercetin, kaempferol, and isorhamnetin in the sea buckthorn juice during 28 d storage. All data were presented as means and standard derivations. Values and error bars represent the means and standard deviations of duplicated experiments (n=3), respectively. The P values were computed by two-tailed Student’s t test (*p < 0.05, **p < 0.01, ***p < 0.001, ****p < 0.0001, ns represents no significance).

**Fig. S3.** The dissolution mechanism of SBF under juicing. A. The external diffusion. B. The internal diffusion. C. The matrix degradation.

**Table captions:**

**Table S1** Concentration of dissolved quercetin, kaempferol, and isorhamnetin of SBF under juicing 1-5 min. Different letters in the same column indicate significant differences (p<0.05).

**Table S2** Colorimetric analysis of juice under 1-5 min juicing. Different letters in the same column indicate significant differences (p<0.05).

**Table S3** Parameters of dissolution kinetic models of quercetin, kaempferol, and isorhamnetin of SBF.


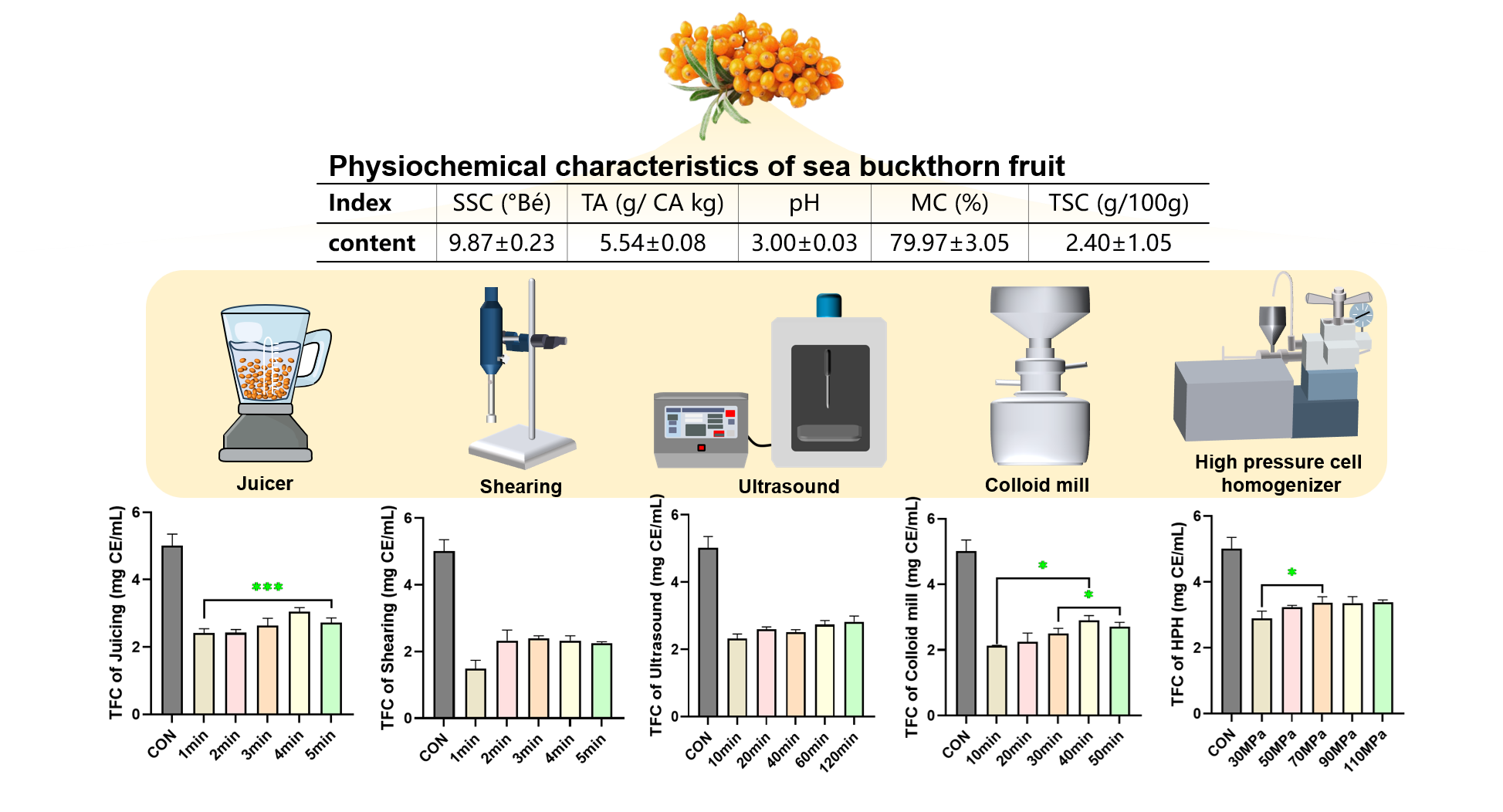


**Fig. S1.** The physiochemical indexes and TFC of SB treated by five non-thermal processing techniques. All data were presented as means and standard derivations. The results were statistically significant (p < 0.05). Values and error bars represent the means and standard deviations of duplicated experiments (n=3), respectively. The P values were computed by two-tailed Student’s t test (*p < 0.05, **p < 0.01, ***p < 0.001, ****p < 0.0001, ns represents no significance).


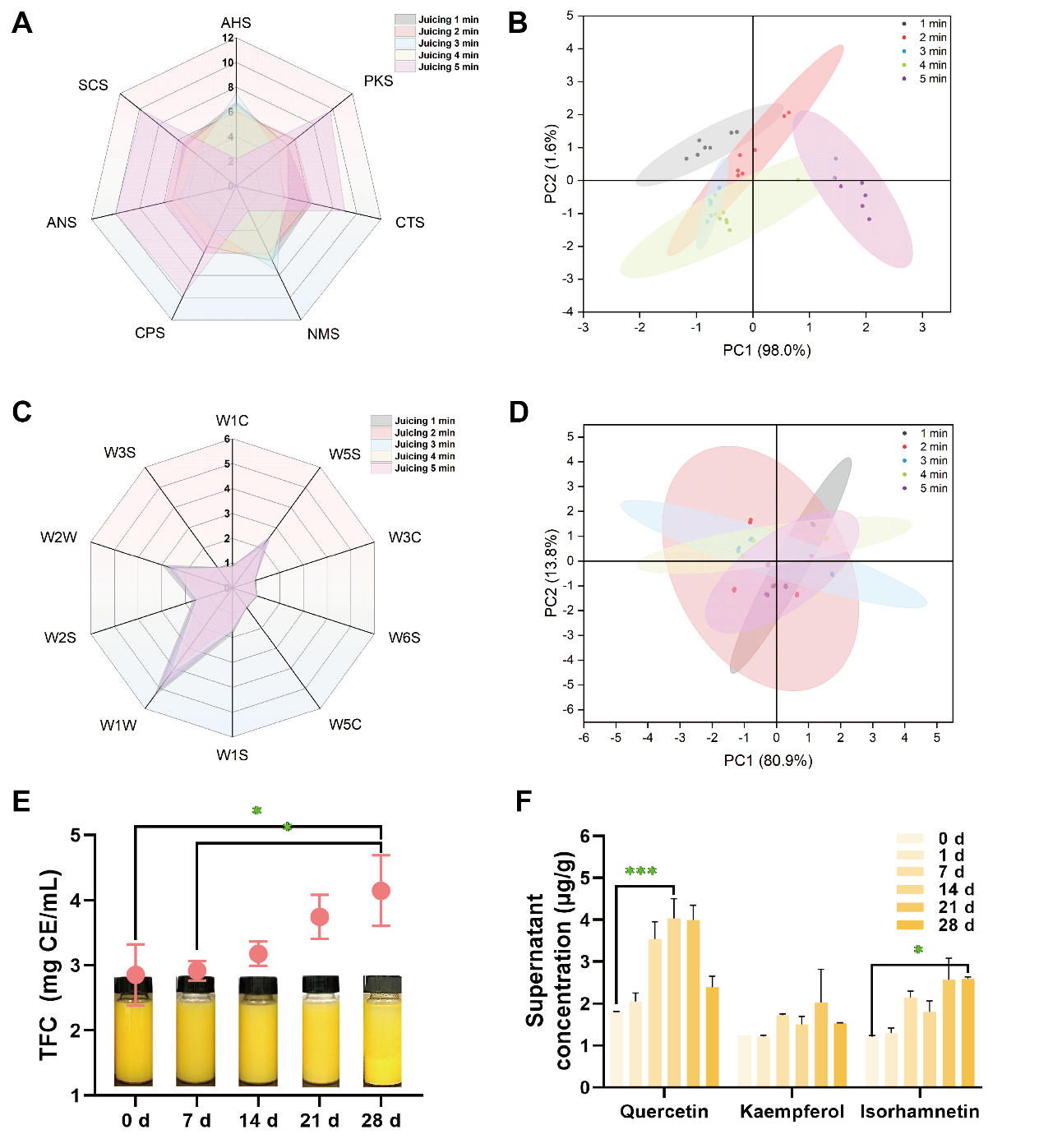


**Fig. S2.** Assessment of electronic senses and storage evaluation in juices. A&B: E-tongue data radar chart and PCA analysis of samples at different juicing time. C&D: E-nose data radar chart and PCA analysis of samples at different juicing time. E: Macroscopic morphology and TFC during 28 d storage. F: Supernatant concentrations of quercetin, kaempferol, and isorhamnetin in the sea buckthorn juice during 28 d storage. All data were presented as means and standard derivations. Values and error bars represent the means and standard deviations of duplicated experiments (n=3), respectively. The P values were computed by two-tailed Student’s t test (*p < 0.05, **p < 0.01, ***p < 0.001, ****p < 0.0001, ns represents no significance).

**
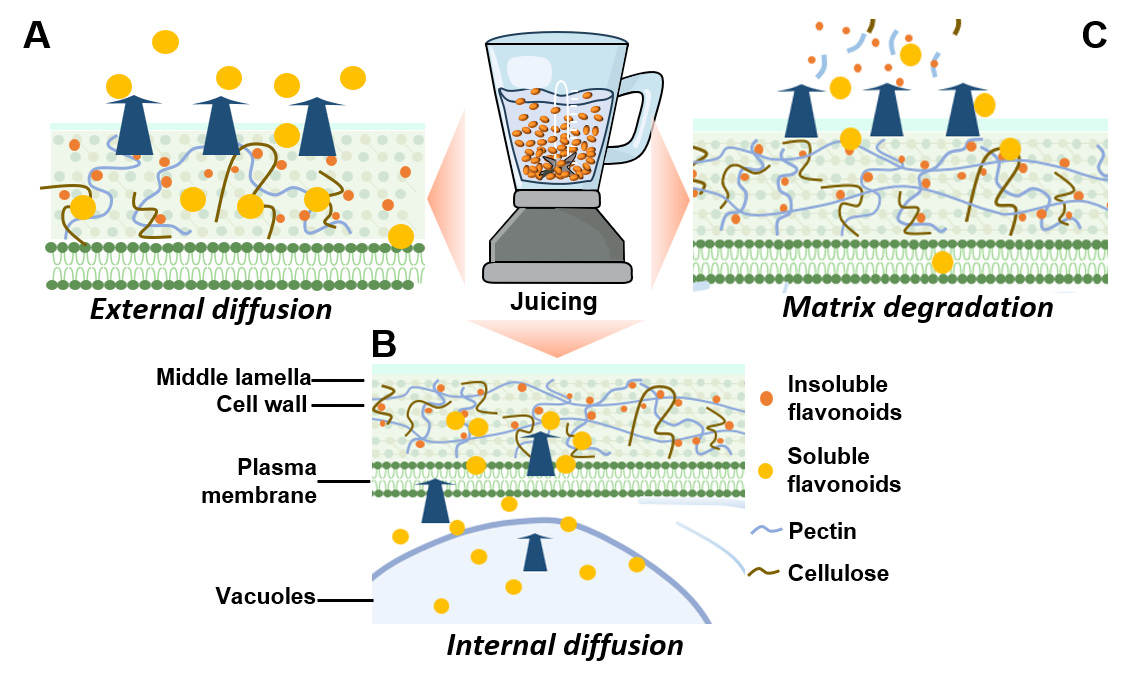
**

**Fig. S3.** The dissolution mechanism of SBF under juicing. A. The external diffusion. B. The internal diffusion. C. The matrix degradation.

**Table S1** Concentration of dissolved quercetin, kaempferol, and isorhamnetin of SBF under juicing 1-5 min.

| Juicing | Flavonol compounds content (μg/g DW) | | | |
| --- | --- | --- | --- | --- |
|  | Quercetin | Kaempferol | | Isorhamnetin |
| 1 min | 1.20±0.16^d^ | 0.00±0.02^c^ | | 0.57±0.07^a^ |
| 2 min | 1.44±0.26^c^ | 0.00±0.02^c^ | | 0.33±0.16^c^ |
| 3 min | 1.48±0.14^c^ | 1.75±0.28^a^ | | 0.63±0.48^a^ |
| 4 min | 1.93±0.21^b^ | 0.88±0.02^b^ | | 0.33±0.13^c^ |
| 5 min | 2.25±0.15^a^ | 0.01±0.01^c^ | | 0.43±0.12^b^ |
| SB | 180.37±15.76^a^ | 124.55±1.86^c^ | | 146.59±6.19^b^ |
| Compounds | Standard curves | | R² | |
| Quercetin | y = 31423x – 83167 | | 0.9998 | |
| Kaempferol | y = 34708x - 157654 | | | 0.9993 |
| Isorhamnetin | y = 44346x - 22916 | | | 1 |

All results are expressed as ‘mean values ± standard deviation’ of three replicates (n = 3). Different letters in the same column indicate significant differences (p<0.05).

**Table S2** Colorimetric analysis of juice under 1-5 min juicing.

| Juicing | L* | a* | b* | ΔE |
| --- | --- | --- | --- | --- |
| 1 min | 50.86±0.57^c^ | 20.29±0.38^a^ | 49.04±0.91^a^ | 46.33±0.23^a^ |
| 2 min | 51.43±0.15^bc^ | 18.81±0.11^b^ | 47.92±0.19^a^ | 44.64±0.04^b^ |
| 3 min | 52.94±0.08^a^ | 19.22±0.07^b^ | 48.89±0.17^a^ | 44.25±0.07^c^ |
| 4 min | 52.39±0.33^ab^ | 18.78±0.29^b^ | 48.18±0.50^a^ | 43.97±0.12^c^ |
| 5 min | 46.37±0.30^d^ | 14.12±0.36^c^ | 40.24±0.55^b^ | 43.47±0.05^d^ |

All results are expressed as ‘mean values ± standard deviation’ of three replicates (n = 3). Different letters in the same column indicate significant differences (p<0.05).

**Table S3** Parameters of dissolution kinetic models of quercetin, kaempferol, and isorhamnetin of SBF.

| Sample | Compound | First-order kinetic model | | Weibull model | | |
| --- | --- | --- | --- | --- | --- | --- |
|  |  | k | R^2^ | k | d | R^2^ |
| 1 min | Quercetin | 0.02 | 0.9792 | 0.03 | 0.94 | 0.9970 |
|  | Kaempferol | ND | ND | ND | ND | ND |
|  | Isorhamnetin | 0.03 | 0.9733 | 0.01 | 1.33 | 0.9879 |
| 2 min | Quercetin | 0.02 | 0.9704 | 0.02 | 1.00 | 0.9937 |
|  | Kaempferol | 0.01 | 0.9242 | 0.01 | 2.73 | 0.9773 |
|  | Isorhamnetin | 0.02 | 0.9789 | 0.02 | 1.01 | 0.9942 |
| 3 min | Quercetin | 0.02 | 0.9773 | 0.02 | 1.04 | 0.9940 |
|  | Kaempferol | 0.02 | 0.9670 | 0.02 | 1.04 | 0.9932 |
|  | Isorhamnetin | 0.03 | 0.9656 | 0.02 | 1.04 | 0.9932 |
| 4 min | Quercetin | 0.03 | 0.9883 | 0.02 | 1.00 | 0.9960 |
|  | Kaempferol | 0.03 | 0.9681 | 0.03 | 0.77 | 0.9958 |
|  | Isorhamnetin | 0.04 | 0.9883 | 0.03 | 0.89 | 0.9960 |
| 5 min | Quercetin | 0.02 | 0.9782 | 0.02 | 1.03 | 0.9950 |
|  | Kaempferol | 0.01 | 0.9865 | 0.01 | 1.23 | 0.9927 |
|  | Isorhamnetin | 0.02 | 0.9920 | 0.02 | 1.04 | 0.9948 |

All results are expressed as ‘mean values ± standard deviation’ of three replicates (n = 3).

ND: incalculable.
